# A New Ursane-Type Nor-Triterpenoid from the Leaves of *Eucommia ulmoides* Oliv.

**DOI:** 10.3390/molecules171213960

**Published:** 2012-11-26

**Authors:** Chuangjun Li, Li Li, Chao Wang, Jingzhi Yang, Fei Ye, Jinying Tian, Yikang Si, Dongming Zhang

**Affiliations:** State Key Laboratory of Bioactive Substance and Function of Natural Medicines, Institute of Materia Medica, Chinese Academy of Medical Sciences and Peking Union Medical College, Beijing 100050, China; E-Mails: lichuangjun@imm.ac.cn (C.L.); annaleelin@imm.ac.cn (L.L.); wangchao@imm.ac.cn (C.W.); yjzh@imm.ac.cn (J.Y.); yefei@imm.ac.cn (F.Y.); tianjinying@imm.ac.cn (J.T.); syk@imm.ac.cn (Y.S.)

**Keywords:** *Eucommia ulmoides* Oliv., nor-triterpenoid, ulmoidol A, absolute configuration, ECD, PTPIB

## Abstract

A new ursane-type nortriterpenoid, (11*S*,12*S*)-4-methyl-11,12-epoxy-2-hydroxy-3-oxoursa-1,4-dine-28-oic acid *γ*-lactone (**1**), named ulmoidol A, together with ten known compounds: ulmoidol (**2**), corosolic acid (**3**), 2*α*,3*α*-dihydroxy-24-nor-4(23),12-oleanadien-28-oic acid (**4**), oleanolic acid (**5**), ursolic acid (**6**), cycloart-3*β*, 25-diol (**7**), foliasalacioside B1 (**8**), (6*R*,7E,9*R*)-9-hydroxy-4,7-megastigmadien-3-one-9-O-*α*-L-arabinopyranosyl-(1→6)-*β*-D-glucopyranoside (**9**), (6*R*,7E,9*R*)-9-hydroxy-4,7-megastigma-dien-3-one-9-*O*-*β*-D-xylopyranosyl-(1→6)-*β*-D-glucopyranoside (**10**), and quercetin 3-*O*-sambubioside (**11**) were isolated from the leaves of *Eucommia ulmoides* Oliv. The structure of compound **1** was determined by extensive spectroscopic analysis, and its absolute configuration was determined by CD experiments and a computational method. Compounds **3**, **4**, **7**–**10** were isolated from this plant for the first time. Compounds **3** and **4** showed inhibition to PTPIB activities, with IC_50_ values of 0.69 and 3.98 μM, respectively.

## 1. Introduction

**Figure 1:**
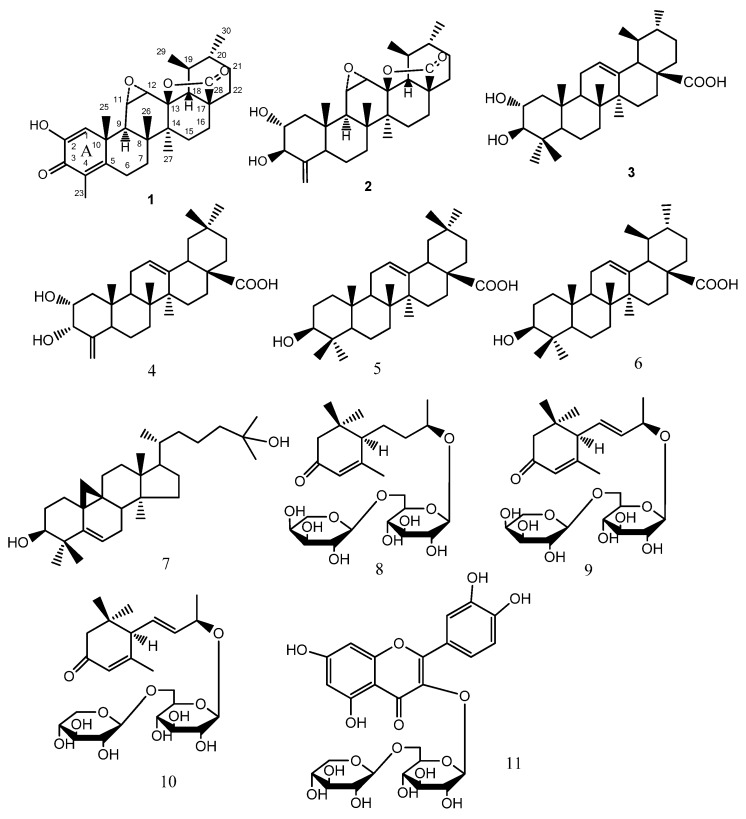
Structures of compounds **1**–**11**.

*Eucommia ulmoides* Oliv. mainly grows along the Yangtze River and in southern China. Its bark has traditionally been applied in China as an antihypertensive, diuretic, sedative, tonic and nourishing agent [1]. The pharmacological effects of *E. ulmoides* are recorded as strengthening the internal organs, bones and muscles, and preventing senescence [2]. The extract of its leaves also showed activities against hypertension and bacteria [2,3]. Previous chemical investigations on this plant have resulted in the isolation of a series of lignanoids [4,5,6,7], phenylpropanolds [8,9,10], iridoids [9,11], flavones [11,12], guttapercha [2], polysaccharides [2], and terpenes [13,14]. As part of a program to study the chemical diversity of traditional Chinese medicines and their biological effects, an ethanol extract of *E. ulmoides* has been investigated. We describe herein the isolation, structure elucidation of a new ursane-type nortriterpenoid, ulmoidol A (**1**), and ten known compounds **2**–**11** from the EtOAc-soluble portion of the ethanol extract of *E. ulmoides* (Figure 1). On the basis of theoretical calculations of its electronic circular dichroism (ECD), the absolute configuration of compound **1** was also established. Compounds **3** and **4** showed inhibition to PTPIB activities with IC_50_ values of 0.69 and 3.98 μM, respectively.

## 2. Results and Discussion

**Figure 2:**
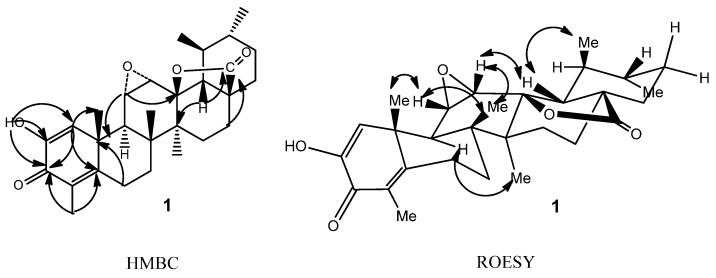
Key HMBC and ROESY correlations of **1**.

**Figure 3:**
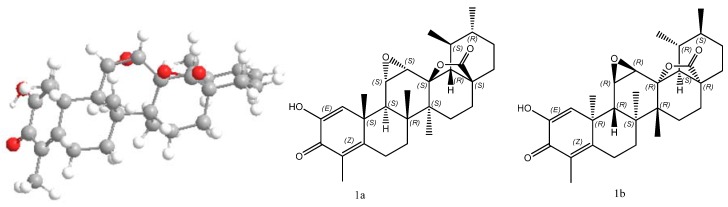
The optimized geometry of compound **1** and two possible structures **1a** and **1b**.

Compound **1** was obtained as a white amorphous powder. Its molecular formula was determined as C_29_H_38_O_5_ by HRESIMS (*m/z* 467.2792, [M+H]^+^), representing 11 degrees of unsaturation. The presence of hydroxyl (3,407 cm^–1^), *γ*-lactone (1,784 cm^–1^) and conjugated ketone (1,618 cm^–1^) groups was evident from its IR spectrum. The ^1^H-NMR spectrum of **1** (Table 1) revealed resonances suggestive of six methyl proton signals at *δ*_H_ 0.97 (3H, s), 0.98 (3H, d, *J* = 5.5 Hz), 1.33 (3H, s), 1.40 (3H, s), 1.18 (3H, d, *J* = 6.5 Hz), and 2.01 (3H, s), two oxygen-bearing methine protons at *δ*_H_ 3.08 (1H, d, *J* = 4.0 Hz) and 3.38 (1H, dd, *J* = 4.0, 2.0 Hz), one olefinic proton at *δ*_H_ 6.47 (lH, brs), and one hydroxyl signal at *δ*_H_ 6.23 (1H, brs). The ^13^C-NMR spectrum revealed 29 carbon resonance signals, including an ester carbonyl (*δ*_C_ 178.83), a conjugated keto carbonyl (*δ*_C_ 181.44), four olefinic carbons (*δ*_C_ 163.62, 144.88, 127.34, and 123.22), and three oxygen-bearing carbons (*δ_C_* 88.67, 54.71 and 54.60). The aforementioned data were indicative of an ursane-type nortriterpenoid skeleton for **1**, similar to those of ulmoidol (**2**) [14]. Compared with compound **2**, the NMR spectral data of compound **1** showed some differences in the A ring, including the absence of two oxygen-bearing carbons and an exo-methylene group, and the presence of a conjugated keto carbonyl, four olefinic carbons and a methyl group, all of which implied the existence of a hexa-1,4-dienone moiety in the A ring of **1**, with the carbonyl at C-3. The above assumption was confirmed by the HMBC correlations of H-1/C-2, C-3, C-5, C-9, C-10 and C-25, and H-23/C-3 and C-5 (Figure 2). The HMBC correlations from *δ*_H_ 6.23 (-OH) to C-1, C-2, C-3 located the hydroxyl group at C-2. The linkage of C-13 and C-28 via a lactone ring was furnished by the carbon chemical shifts of C-18 and C-28, as well as the key HMBC correlations between H-18 and C-13, C-14, and C-28; H-22/C-17 and C-18 and H-16/C-17 and C-28. The HMBC correlations of H-12/C-13 and C-11; H-11/C-10 and C-9 verified the three-member epoxy group at C-11 and C-12, as showed in compound **2**. Moreover, the key ROESY correlations of H-11, H-12/H_3_-26; H-11/H-25 suggested the *α*-orientation of the 11,12-epoxide (Figure 2). The relative configuration of **1** was deduced according to ROESY experiment and the literature [14,15], leading to two possible structures **1a** and **1b** (Figure 3). 

**Figure 4:**
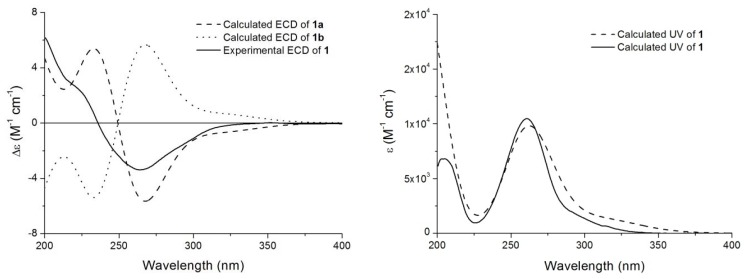
Comparison of theoretical and experimental ECD and UV spectra of compound **1**.

The absolute configuration of compound **1** was established by theoretical calculation of its electronic circular dichroism (ECD) using the time-dependent density functional theory (TD-DFT) method [16]. Their optimized geometries were obtained, and then the ECD spectra were calculated at the B3LYP/6-31G(d) level with the TD-DFT/PCM model in methanol solution [17]. As shown in Figure 4, the calculated ECD spectrum of **1a** exhibited a diagnostic negative Cotton effect at around 252 nm, corresponding to the experimental Cotton effect observed at 254 nm. Therefore, the absolute configuration of compound **1** was determined as (11*S*,12*S*)-4-methyl-11,12-epoxy-2-hydroxy-3-oxoursa-1,4-dine-28-oic acid *γ*-lactone (**1a**), and the compound was named ulmoidol A.

Ten known compounds, namely ulmoidol (**2**) [14], corosolic acid (**3**) [18], 2*α*,3*α*-dihydroxy-24-nor-4(23), 12-oleanadien-28-oic acid (**4**) [19], oleanolic acid (**5**) [20], ursolic acid (**6**) [18], cycloart-3*β*, 25-diol (**7**) [21], foliasalacioside B1 (**8**) [22], (6*R*,7*E*,9*R*)-9-hydroxy-4,7-megastigmadien-3-one-9-*O*-*α*-L-arabinopyranosyl-(1→6)-*β*-D-glucopyranoside (**9**) [23], (6*R*,7*E*,9*R*)-9-hydroxy-4,7-megastigmadien-3-one-9-O-*β*-D-xylopyranosyl-(1→6)-*β*-D-glucopyranoside (**10**) [24], and quercetin 3-*O*-sambubioside (**11**) [11] were also identified on the basis of their spectroscopic profiles (NMR, UV, CD and MS) and comparisons to published data.

**Figure 5:**
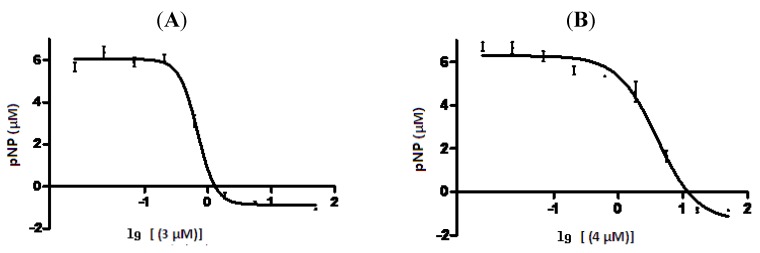
Inhibition of Compounds **3** (**A**) and **4** (**B**) on PTP1B activity *in vitro*.

The *in vitro* inhibition of protein tyrosine phosphatase 1B (PTP1B) activity by compounds **1**–**6**, **8**–**10** was tested at 10 μM*,* with the known effective compound CCCF06240 as positive control [25]. As shown in Table 2, compounds **3** and **4** exhibited inhibition to PTPIB activities, with IC_50_ values of 0.69 and 3.98 μM, respectively (Figure 5).

## 3. Experimental

### 3.1. General Procedures

Optical rotations were measured on a P2000 automatic digital polarimeter. UV spectra were taken with a Hitachi UV-240 spectrophotometer. CD spectra were measured on a JASCO *J*-815 spectro-polarimeter. IR spectra were recorded on a Nicolet 5700 FT-IR spectrometer. NMR measurements were performed on INOVA-500 and Bruker AV500-III spectrometers. HRESIMS were obtained using an Agilent 1100 series LC/MSD Trap SL mass spectrometer. Preparative HPLC was carried out on a Shimadazu LC-6AD instrument with a SPD-20A detector, using a YMC-Pack ODS-A column (250 × 20 mm, 5 µm). Column chromatography (CC) was performed with silica gel (200–300 mesh, Qingdao Marine Chemical Inc., Qingdao, People’s Republic of China) and ODS (50 µm, YMC, Tokyo, Japan). TLC was carried out with glass precoated silica gel GF_254_ plates. 

### 3.2. Plant Material

The leaves of *E. ulmoides* were collected in Liuzhou, Guangxi, China, in August 2008, and identified by Associate Professor Lin Ma of the Institute of Materia Medica, Chinese Academy of Medical Science, Beijing, China. A voucher specimen had been deposited at the Herbarium of Institute of Materia Medica, Chinese Academy of Medical Sciences & Peking Union Medical College (200816).

### 3.3. Extraction and Isolation

The dried leaves of *E. ulmoides* (2.0 kg) were powdered and extracted with 95% ethanol (30 L × 3) under reflux. The filtrate was evaporated under reduced pressure to yield a dark brown residue (320 g). The residue was suspended in water (2,000 mL) and then successively partitioned with EtOAc (3 × 1,000 mL) and *n*-BuOH (3 × 1,000 mL). After removing the solvent, the EtOAc-soluble portion (120 g) was fractionated via silica gel CC eluting with a CHCl_3_-MeOH gradient (100:0–3:1) to afford ten fractions A_1_-A_10_ on the basis of TLC analysis. Fraction A_2_ (16.301 g) was chromatographed over silica gel (200–300 mesh) eluted with a CHCl_3_-MeOH gradient (100:1, 50:1) to give **2** (25 mg), **3** (8 mg), **4** (8 mg), **5** (450 mg), **6** (360 mg), and **7** (9 mg) and a mixture (20 mg). This mixture was further purified by preparative HPLC on ODS (YMC-pack; eluent: MeCN-H_2_O, 30:70, v/v, 7 mL/min, 210 nm) to afford **1** (t_R_ 54 min, 8 mg). Fraction A_7_ (8.48 g) was chromatographed over silica gel (200–300 mesh) eluted with a CHCl_3_-MeOH gradient (20:1, 8:1, 4:1) to give nine subfractions, A_7_a–A_7_i. Fraction A_7_e was purified by preparative HPLC (YMC-pack; eluent: MeOH-H_2_O, 35:65, v/v, 7 mL/min, 254 nm) to yield **11** (t_R_ 37 min, 15 mg). Fraction A_7_f was purified by preparative HPLC (YMC-pack; eluent: MeOH-H_2_O, 50:50, v/v, 7 mL/min, 210 nm) to yield **8** (t_R_ 45 min, 10 mg), **9** (t_R_ 55 min, 9 mg), and **10** (t_R_ 67 min, 7 mg).

### 3.4. Spectral Data

*Ulmoidol A* (**1**). White amorphous powder, ${\left[\alpha \right]}_{D}^{20}$ + 7.56 (*c* 0.08, MeOH); UV (MeOH) *λ*_max_ (log *ε*) 208 (2.54), 258 (4.21) nm; IR *ν*_max_ cm^−1^: 3,407, 2,935, 1,784, 1,618, 1,241, and 937. ESIMS *m/z*: 489 [M+Na]^+^, 467 [M+H]^+^; HRESIMS *m/z*: 467.2804 [M+H]^+^ (calcd. 467.2792, C_2_α_9_H_39_O_5_). For ^1^H and ^13^C-NMR spectroscopic data, see Table 1.

### 3.5. PTP1B Activity Assay *[25]*

Recombinant human GST-PTP1B protein was overexpressed by hGST-PTP1B-BL21 *E. coli* and purified by GST affinity chromatography. The reagent pNPP was used as substrate for the measurement of PTP1B activity. Compounds were pre-incubated with the enzyme at room temperature for 5 min. Assay was performed in final volume of 100 μL in the active system containing 50 Mm HEPES, 5 mM DTT, 150 mM NaCl, 2 mM EDTA, and 2 mM pNPP (pH 7.0), incubated at 30 °C for 10 min, stopped by addition of 50 μl 3 M NaOH. Then, the absorbance was determined at 405 nm wavelength. The similar system without GST-PTP1B protein was used as blank. The effects of compounds **1**–**6**, **8**–**10** on PTP1B activity were measured, and the IC50 value was calculated by nonlinear regression.

## 4. Conclusions 

A new ursane-type nortriterpenoid, ulmoidol A (**1**), together with ten known compounds were isolated from the leaves of *Eucommia ulmoides* Oliv. The structure of compound **1** was determined by extensive spectroscopic analysis, and the absolute configuration was determined by CD experiments and computational methods. Compounds **3**, **4**, **7**–**10** were isolated from this plant for the first time. Compounds **1**–**6**, and **8**–**10** were tested for inhibition of PTP1B activities, and compounds **3** and **4** showed inhibit activities with IC_50_ values of 0.69 and 3.98 μM, respectively.

## Figures and Tables

**Table 1:** NMR data of compound **1** in CDCl_3_ (500 MHz for ^1^H-NMR and 125 MHz for ^13^C-NMR).

Position	^1^H	^13^C
1	6.47 s	123.22
2		144.88
2-OH	6.23 s	
3		181.44
4		127.34
5		163.62
6	2.50 (td, 13.5, 4.5) 2.80 (brd, 13.5)	24.10
7	1.39 m 1.26 m	31.93
8		41.68
9	1.75 (brs)	47.72
10		41.91
11	3.38 (dd, 4.0, 2.0)	54.71
12	3.08 (d, 4.0)	56.60
12		
13		88.67
14		41.68
15	1.11(dd,14.0,5.5) 1.74 m	27.20
16	2.13 (td, 13.5, 5.5) 1.37 m	22.68
17		45.10
18	1.79 m	60.46
19	0.97 m	40.20
20	1.67m	37.50
21	1.60 m1.25 m	30.49
22	1.81 m 1.53 (td, 13.5, 4.5)	31.32
23	2.01 s	10.89
25	1.40 s	22.44
26	1.33 s	19.13
27	0.97 s	16.21
28		178.83
29	1.18 (d, 6.5)	17.16
30	0.98 (d, 5.5)	19.46

**Table 2:** The inhibition to PTP1B activities of compounds **1**–**6**, **8**–**10**.

Comp.	Inhibition (%) at 10 μM
1	4.8
2	3.0
3	81.3
4	79.8
5	28.3
6	43.5
8	13.4
9	28.6
10	17.8
CCCFO6240 ^a^	108.5

^a^ Positive control.
